# Interpretable Fault Diagnosis of Shearer Power-Core Cables from Sensor-Accessible Terminal Electrical Responses

**DOI:** 10.3390/s26185968

**Published:** 2026-09-21

**Authors:** Lijuan Zhao, Jiazheng Bu, Beichen Jiang, Tiangu Wu

**Affiliations:** School of Mechanical Engineering, Liaoning Technical University, Fuxin 123000, China

**Keywords:** shearer trailing cable, terminal electrical response, distributed-parameter model, categorical Mamdani inference, grouped validation, interpretable fault diagnosis

## Abstract

Shearer trailing cable faults are difficult to distinguish from terminal measurements because their signatures coexist with changes in load, source imbalance, cable temperature, and sensor error. This study presents a physics-guided categorical Mamdani framework supported by a distributed-parameter source, cable, and load model. A total of 180 simulations cover normal operation, phase-to-ground faults, A–B inter-phase short circuits, conductor-resistance degradation, and insulation-path deterioration over multiple severities, locations, motor loads, source imbalance levels, and temperatures. Paired pre-fault and post-fault changes in load-terminal voltage unbalance, source-terminal zero-sequence current ratio, and source-to-load voltage attenuation are mapped to fault path-based membership functions and diagnostic rules. Twenty repeated 70/30 holdouts grouped by base operating condition compare the proposed method with a deterministic threshold tree, radial-basis-function support vector machine, and k-nearest-neighbor classifier. Across these repeated grouped holdouts, the proposed method achieves 99.80% mean accuracy with a standard deviation of 0.63 percentage points on noise-free test cases. Accuracy remains 81.12% and 74.71% under 1% and 2% RMS waveform noise, respectively. These results demonstrate the effectiveness of the proposed framework for interpretable diagnosis of shearer power-core cable faults across the investigated simulated operating conditions and waveform-noise levels.

## 1. Introduction

Power cables in mobile mining equipment operate under a combination of electrical loading and repeated mechanical disturbance. Dragging, bending, vibration, moisture ingress, coal-dust contamination, and thermal cycling progressively alter conductor continuity and insulation condition. Recent reviews have summarized advances and remaining challenges in power-cable aging diagnosis [1], while material-oriented studies have examined condition assessment and service-life prediction for low-voltage cross-linked polyethylene cables [2]. For trailing cables, these degradation processes must be interpreted within a moving service environment and a coupled source-load system.

Existing cable diagnostics can be broadly divided into insulation-discharge monitoring and traveling-wave analysis. Temperature-dependent discharge observations and terminal-condition reviews illustrate the value of discharge and structural indicators [3,4], while partial-discharge theory, localization circuits, and single-ended methods emphasize high-frequency signal acquisition [5,6,7]. Traveling-wave approaches likewise provide effective fault detection and localization [8,9]. These techniques are important for high-voltage fault analysis but do not directly address the power-frequency terminal responses already available in a moving shearer supply system.

Research closer to the mining application has examined the reliability of drum-shearer cable systems and multiparameter cable assessment [10,11]. Distributed optical-fiber, acoustic, and strain sensing provide valuable spatial information [12,13,14], whereas deep learning and convolutional recognition can identify complex discharge patterns [15,16]. However, these approaches either require additional sensing infrastructure or representative labeled fault data, both of which are difficult to obtain for destructive shearer-cable faults.

The service characteristics of shearer cables intensify this limitation. Dynamic analysis has revealed repeated excitation within the cable-drag system [17], and electromagnetic-thermal-mechanical studies have shown interaction between electrical and structural states [18]. Safety analyses have also established the operational consequences of trailing-cable faults in mobile mining equipment [19]. A practical diagnostic method should therefore separate fault-related changes from load, source, and temperature effects while retaining an inspectable decision process.

Fuzzy inference provides a basis for such an interpretable decision layer. Fuzzy-set theory enables gradual transitions between linguistic states [20], and adaptive-network-based fuzzy inference combines reasoning with parameter learning [21]. Self-organizing fuzzy models have extended this concept to semi-supervised and evolving data streams [22], while domain-adaptation methods demonstrate the importance of performance under changing operating distributions [23]. For cable monitoring, fuzzy reasoning can express fault mechanisms through traceable memberships and rule activation.

A gap therefore remains between high-frequency fault-detection methods and the power-frequency measurements routinely available at the source and motor terminals. The central question is whether compact terminal-response features can separate representative conductive and insulation-path disturbances across variable operating conditions while retaining physically interpretable boundaries. This requirement is particularly relevant where electrical apparatus must satisfy the safety provisions for explosive gas atmospheres [24].

This study develops an application-specific, physics-guided framework for shearer power-core cables by combining a distributed-parameter source–cable–load model with categorical Mamdani-style inference. Its contribution lies in a fault path-oriented mapping from paired terminal-response changes to transparent diagnostic rules, together with grouped holdout evaluation across withheld combinations of motor load, source imbalance, and cable temperature generated within the same simulation framework. The 180-case investigation includes normal operation and four equivalent fault classes over multiple severities and locations. The proposed method is compared with a threshold tree, an RBF-SVM, and a k-NN classifier under waveform-level measurement noise.

## 2. Distributed-Parameter Source–Cable–Load Model and Reproducible Case Design

The model comprises a programmable three-phase source, a 1 MVA transformer, identical phase-interface impedances, two distributed-parameter cable sections, a 730 kW asynchronous motor, and an auxiliary terminal load. The 100 m cable is partitioned at the prescribed fault location so that the source and load boundary conditions remain unchanged across all cases.

The power path is parameterized as a 660 V mining trailing-cable system. Positive- and zero-sequence line parameters govern attenuation, phase displacement, and sequence-component propagation along the cable. The numerical component parameters are summarized in Table 1.

**Figure 1:**
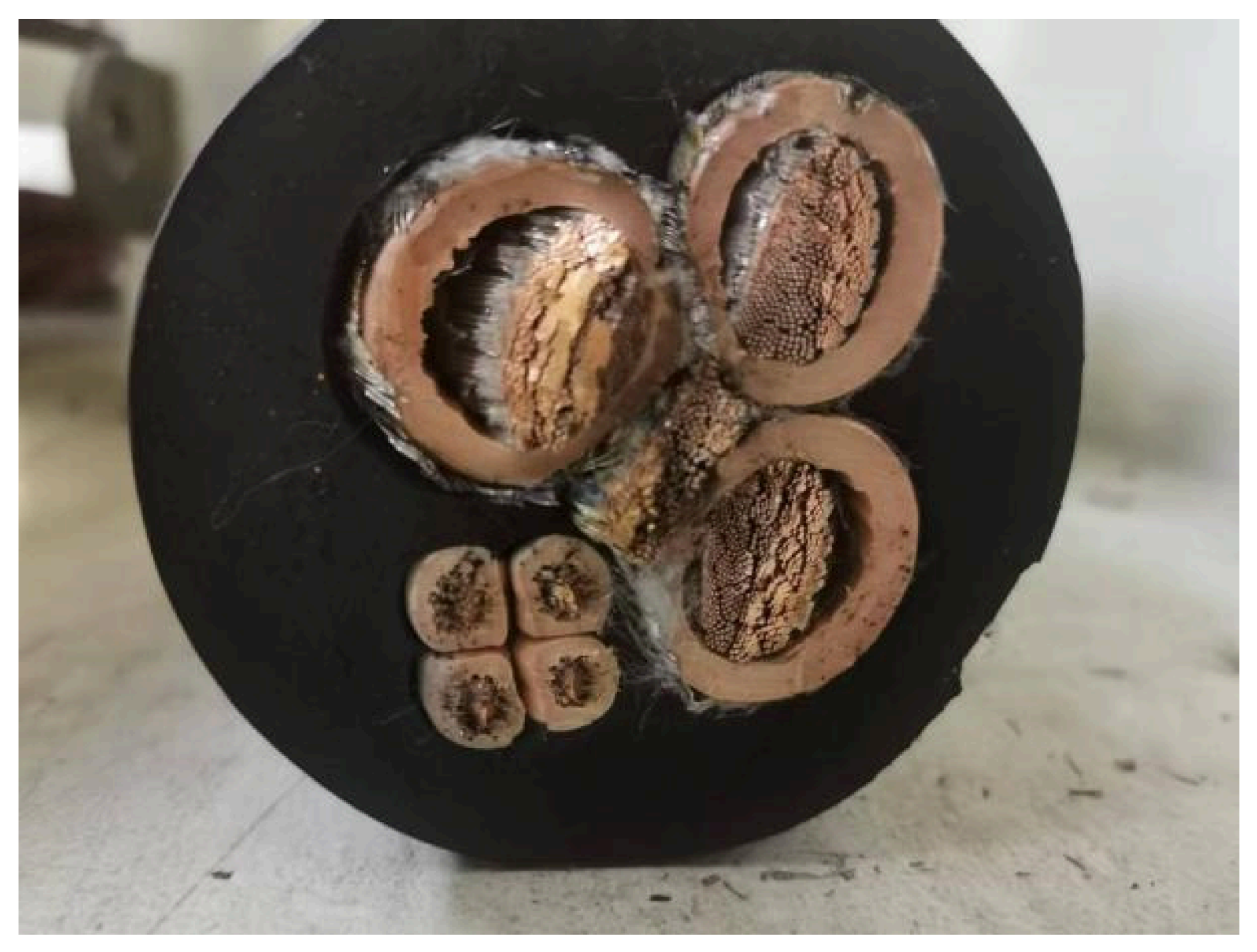
Cross-sectional structure of the cable.

The reference cable cross-section is shown in Figure 1. Laboratory photographs and DC resistance measurements were used to define its geometry and a physically representative range of conductor degradation. Terminal electrical responses under energized fault conditions were subsequently generated using the source–cable–load model.

### 2.1. Distributed-Parameter Basis and Theoretical Foundation

The power-core model is established on the basis of distributed-parameter transmission-line theory and multi-conductor electromagnetic coupling. The three power cores are represented as mutually coupled phase conductors transmitting electrical power from the source side to the asynchronous motor load.

Each conductor is described by per-unit-length resistance, inductance, conductance, and capacitance, while self- and mutual-coupling effects are represented by the corresponding parameter matrices **R**, **L**, **G**, and **C**. This formulation characterizes voltage and current propagation, attenuation, and phase shift along the cable under the combined influence of conductor loss and inter-core coupling.

The parameter setting and boundary conditions are specified in accordance with the structural and electrical characteristics of mining trailing cables, so that the conductor arrangement, dielectric environment, dynamic response, and numerical stability of the source–cable–load transmission path are preserved under power-frequency operation.

The coupled propagation of voltage and current along the power cores satisfies the multi-conductor transmission-line equation

(1) $$\begin{array}{rr} \frac{\partial V(x,t)}{\partial x} & =-\mathrm{RI}(x,t)\;-\;L\frac{\partial I(x,t)}{\partial t},\\ \frac{\partial I(x,t)}{\partial x} & =-\mathrm{GV}(x,t)\;-\;C\frac{\partial V(x,t)}{\partial t}. \end{array}$$

which provides the theoretical basis for analyzing the time-domain terminal electrical responses of the system under normal and faulted conditions.

### 2.2. Source–Cable–Load Configuration

**Figure 2:**
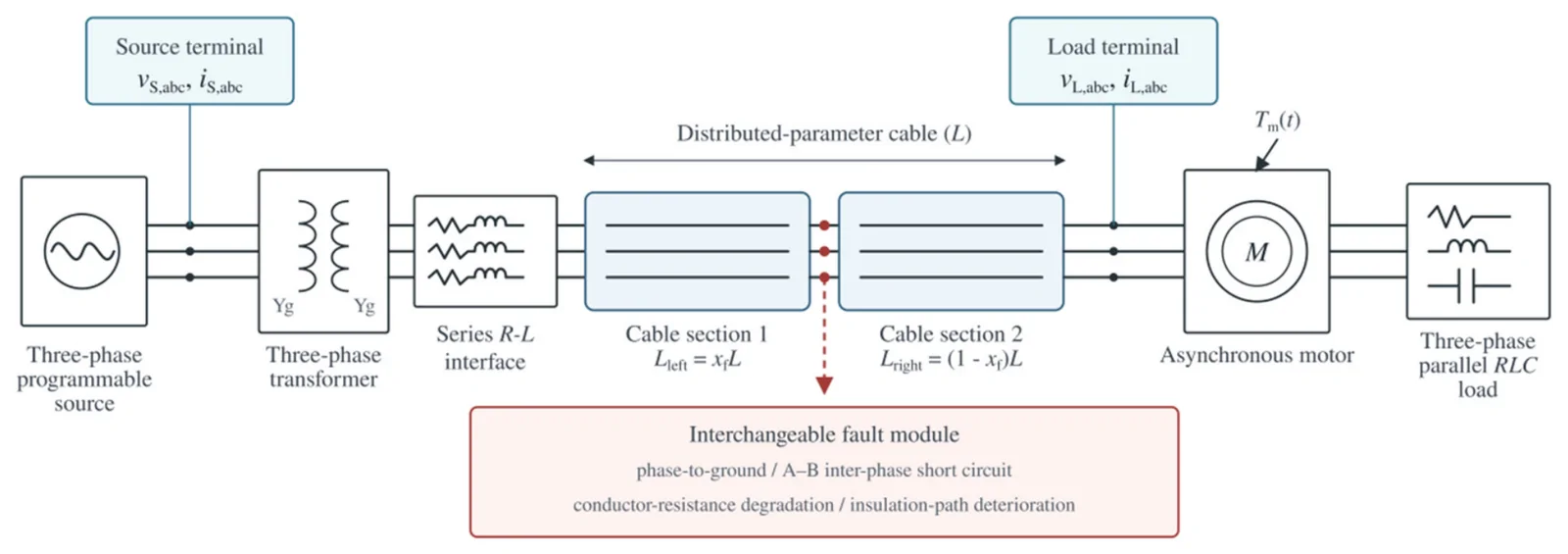
Source-cable-load topology and terminal measurement locations.

Figure 2 shows the complete topology and terminal measurement locations. The cable is divided into left and right distributed sections at the fault insertion node, and three-phase voltage and current are acquired at both terminals.

The programmable source feeds the asynchronous motor through a grounded-wye transformer, identical per-phase interface impedances, and two distributed-parameter cable sections. Source- and load-terminal voltages and currents are measured at the locations indicated in Figure 2, while the interchangeable fault module is inserted only at the cable partition node. The component equations are presented in Section 2.3, and the numerical parameters are summarized in Table 1.

This configuration establishes a common transmission path for all operating and fault cases. Fault modules are inserted only at the cable partition node, leaving the source, transformer, and load definitions unchanged.

### 2.3. Component Equations and Equivalent Parameters

The electrical and mechanical components shown in Figure 2 are parameterized using the relationships below. Table 1 consolidates the numerical inputs common to all simulations.

(1) Three-phase source

Under nominal conditions, the programmable source produces a balanced, power-frequency three-phase voltage set:

(2) $$\begin{array}{cc} u_{a}(t) & =U_{m}\sin(\omega t),\\ u_{b}(t) & =U_{m}\sin\left(\omega t-\frac{2\pi }{3}\right),\\ u_{c}(t) & =U_{m}\sin\left(\omega t+\frac{2\pi }{3}\right), \end{array}$$

In Equation (2), U_m_ and ω denote the phase-voltage amplitude and angular frequency, respectively. The specified line-to-line RMS voltage is 660 V and ω = 2π × 50 rad/s. Source imbalance is introduced by reducing only the phase-A voltage amplitude by 0%, 1%, or 2%, while the remaining source quantities are unchanged.

(2) Transformer

A grounded-wye to grounded-wye two-winding transformer couples the programmable source to the cable. Using the three-phase apparent-power base *S*_b_ and line-to-line voltage base *U*_b_, the corresponding base impedance and line current are

(3) $$Z_{b}=\frac{U_{b}^{\;2}}{S_{b}},\;I_{b}=\frac{S_{b}}{\sqrt{3}\;U_{b}},$$

The associated per-unit normalization is written as

(4) $$R_{\mathrm{pu}}=\frac{R}{Z_{b}},\;X_{\mathrm{pu}}=\frac{X}{Z_{b}},\;B_{m,\mathrm{pu}}=B_{m}Z_{b},$$

In Equation (4), *R* and *X* denote winding resistance and leakage reactance, respectively, and *B*_m_ denotes magnetizing susceptance. In the implemented transformer block, the parameters are entered directly in per-unit form on a 1 MVA, 660 V, and 50 Hz base. Both grounded-wye windings use *R*_1_ = *R*_2_ = 0.002 p.u. and *L_ℓ_*_1_ = *L_ℓ_*_2_ = 0.08 p.u.; the magnetizing branch is defined by *R*_m_ = 1000 p.u. and *L*_m_ = 1000 p.u. The core is represented by three single-phase transformer units, the zero-sequence flux-path inductance is *L*_0_ = 0.5 p.u., and magnetic saturation is disabled.

(3) Series interface branches

Three identical series RL branches represent the per-phase interface impedance between the transformer and the distributed-parameter cable:

(5) $$Z_{s}(\omega )\;=\;R_{s}\;+\;j\omega L_{s},$$

The parameters are *R*_s_ = 0.05 Ω and *L*_s_ = 1 mH per phase. No series capacitance is included in these branches, and the same values are applied to phases A, B, and C.

(4) Distributed-parameter cable

Consistent with the multiconductor formulation in Equation (1), the per-unit-length series-impedance and shunt-admittance matrices are

(6) Z=R+jωL, Y=G+jωC,

where **R**, **L**, **G**, and **C** denote the per-unit-length resistance, inductance, conductance, and capacitance matrices, respectively. For a cable segment of length *l*, the terminal-state relation is

(7) $$\left[\begin{array}{c} V(x+l)\\ I(x+l) \end{array}\right]=T(l)\left[\begin{array}{c} V(x)\\ I(x) \end{array}\right],$$

In Equation (7), T(l) denotes the transmission operator, whereas Z and Y are the per-unit-length series-impedance and shunt-admittance matrices, respectively. The 0.100 km cable is divided at the normalized fault position $x_{f}$, giving $L_{\mathrm{left}}\;={\;x}_{f}\;L$ and $L_{\mathrm{right}}\;=\;(1-x_{f})L$. Thus, $(x_{f}\;\;=\;0.25)$, 0.50, and 0.75 represent the source-side, midpoint, and load-side fault locations, respectively.

Each section is implemented as a three-phase Distributed Parameters Line at 50 Hz and is specified directly in the sequence domain by *R*_seq_ = [0.02, 0.05] Ω/km, *L*_seq_ = [1.0, 0.5] mH/km, and *C*_seq_ = [1, 10] μF/km; the first and second entries are the positive- and zero-sequence quantities, respectively, and the shunt conductance is set to zero. Cable temperature affects the resistance according to

(8) $$R_{\mathrm{seq}}(T)=R_{\mathrm{seq}}(20\;{}^{\circ}C)[1+\alpha (T-20\;{}^{\circ}C)],$$

with *α* = 0.00393 °C^−1^, whereas *L*_seq_ and *C*_seq_ remain fixed. The line block internally converts these sequence inputs for the three-phase solution; no independent phase-domain matrices are assigned in the implementation.

(5) Asynchronous motor

The rated motor current is related to the nominal quantities by

(9) $$I_{N}=\frac{P_{N}}{\sqrt{3}\;U_{N}\;\eta \cos\varphi },$$

where *P*_N_ is the rated power, *U*_N_ is the rated line voltage, *η* is the efficiency, and cos *φ* is the power factor. The motor block is parameterized directly in SI units; its electrical and mechanical parameter sets are denoted by

(10) $$\Theta_{e}=\{R_{s}^{\left(m\right)},\;R_{r}^{\prime (m)},\;L_{s}^{\left(m\right)},L_{r}^{\prime (m)},\;L_{m}^{\left(m\right)}\},$$

and

(11) $$\Theta_{m}=\{J,\;B,\;T_{L}\},$$

The wound-rotor asynchronous motor is rated at 730 kW, 660 V, and 50 Hz. Its parameters are *R_s_*^(*m*)^ = 0.035 Ω, *L_ℓs_*^(*m*)^ = 1 mH, *R_r_^’^*^(*m*)^ = 0.015 Ω, *L_ℓr_^’^*^(*m*)^ = 1.2 mH, and *L_m_*^(*m*)^ = 0.06 H, with *J* = 10 kg·m^2^, *B* = 0.005879 N·m·s, and two pole pairs. The applied mechanical torque *T_L_* is varied among 100, 250, and 400 N·m to represent the three investigated loading levels.

(6) Terminal parallel RLC load

A grounded-wye three-phase parallel RLC branch represents the auxiliary terminal load. The block operates in constant-PQ mode at 660 V and 50 Hz. For the per-phase active power *P_φ_* and reactive power *Q_φ_*, the equivalent admittance is

(12) $$Y_{\phi }=\frac{P_{\phi }}{U_{\phi }^{2}}+j\frac{Q_{C,\phi }-Q_{L,\phi }}{U_{\phi }^{2}},$$

where $U_{\phi }$ is the phase RMS voltage, and $Q_{L,\phi }$ and $Q_{C,\phi }$ denote the positive magnitudes of the inductive and capacitive reactive powers assigned to phase ϕ, respectively. The corresponding resistance, inductance, and capacitance are obtained as

(13) $$R_{\phi }=\frac{U_{\phi }^{2}}{P_{\phi }},\;L_{\phi }=\frac{U_{\phi }^{2}}{\omega Q_{L,\phi }},\;C_{\phi }=\frac{Q_{C,\phi }}{\omega U_{\phi }^{2}},$$

In the balanced auxiliary-load setting, each phase is assigned an active power of 10 kW and inductive and capacitive reactive powers of 0.1 kvar each. The equal inductive and capacitive components yield zero net reactive power at the nominal operating point.

Numerical simulations were conducted in MATLAB R2024b/Simulink using Specialized Power Systems. The continuous ode23tb solver was adopted with a maximum integration step of 10 μs. Each simulation covered 2.5 s, with fault inception specified at 1.5 s. The twelve source- and load-terminal voltage and current channels were sampled at 10 kHz. Feature extraction was performed over the steady-state pre-fault and post-fault intervals of 1.2–1.4 s and 2.2–2.4 s, respectively, excluding startup and fault-inception transients.

## 3. Fault Representation and Terminal Feature Extraction

The source–cable–load configuration provides a common electrical reference for distinguishing fault-induced changes at the accessible terminals. Representative cable defects are therefore introduced as localized impedance modifications at the interconnection node between the two cable sections. The resulting source- and load-terminal waveforms are then processed over matched pre- and post-fault windows to obtain physically interpretable indicators.

### 3.1. Representative Fault Mechanisms

Four fault classes are considered: an A-phase-to-ground fault, an A–B inter-phase short circuit, local conductor-resistance degradation, and insulation-path deterioration. These classes represent, respectively, a phase-to-ground conductive path, an inter-phase conductive bridge, increased series impedance caused by strand or joint damage, and deterioration of the phase-to-ground dielectric path.

Ground and inter-phase faults primarily redistribute the sequence components of the terminal voltages and currents, whereas local conductor degradation increases the phase-specific voltage drop along the cable. Insulation-path deterioration produces comparatively weaker leakage and phase-path changes. The latter is treated as a power-frequency equivalent at the terminal scale and is not intended to reproduce the stochastic high-frequency pulses associated with partial-discharge activity.

### 3.2. Construction and Parameterization of the Fault Modules

Each fault module is inserted at the cable partition node and activated at *t_f_* = 1.5 s. Three normalized fault locations, *x_f_* = 0.25, 0.50, and 0.75, are considered to account for the position-dependent impedance between the fault and the two measurement terminals.

(1) phase-to-ground fault

**Figure 3:**
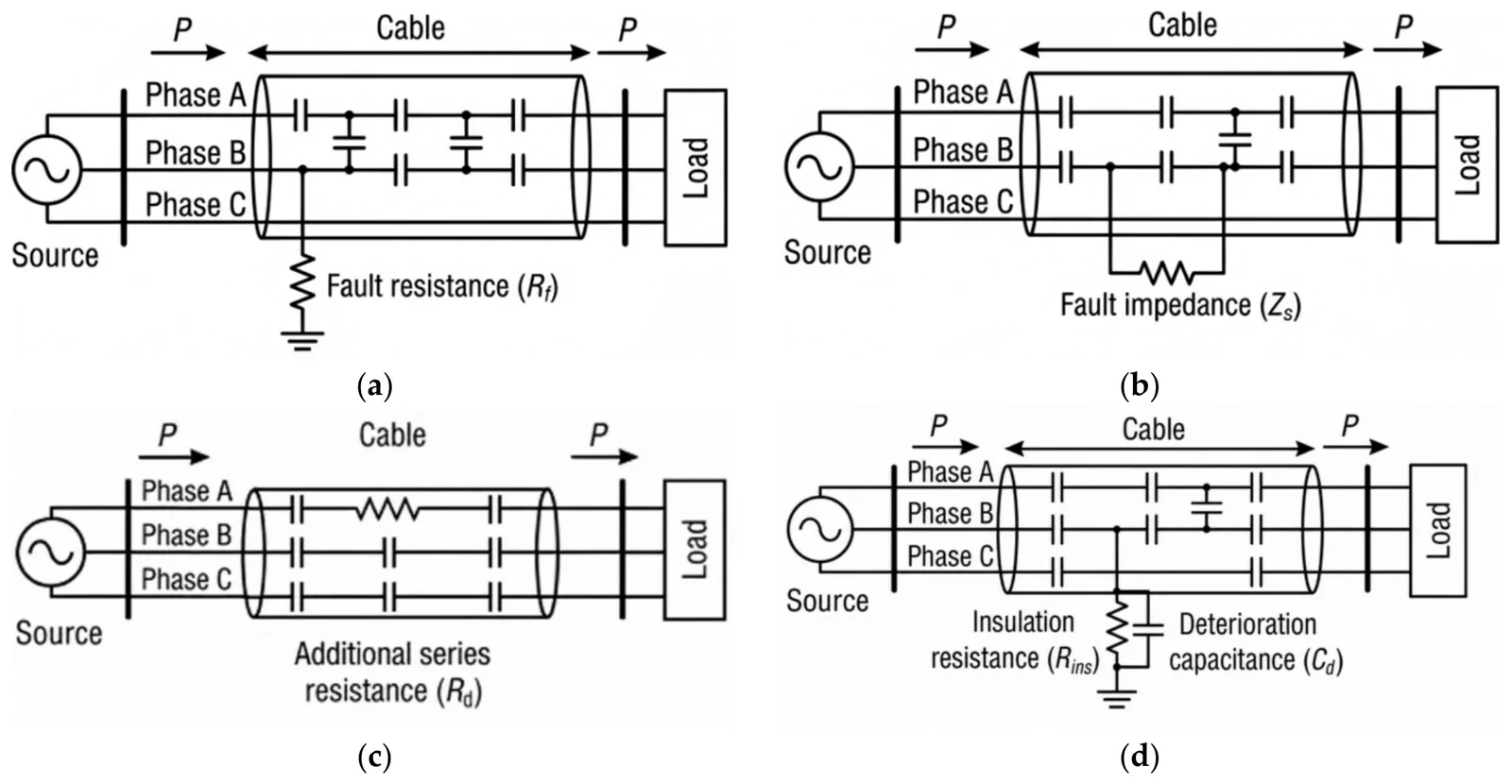
Equivalent fault modules: (**a**) A-phase-to-ground fault; (**b**) A–B inter-phase short circuit; (**c**) local conductor-resistance degradation; (**d**) insulation-path RC deterioration.

The A-phase conductor is connected to ground through a finite fault resistance *R_f_*, as illustrated in Figure 3a. Four values (2, 1, 0.5, and 0.2 Ω) represent progressively stronger phase-to-ground conduction as the fault resistance decreases.

(2) A–B inter-phase short circuit

A resistive branch with impedance *Z_s_* is connected between phases A and B (Figure 3b). The four values of 0.2, 0.1, 0.05, and 0.02 Ω describe increasing degrees of conductive bridging between adjacent phase conductors.

(3) Local conductor-resistance degradation

Local strand or joint damage is represented by an additional series resistance in phase A (Figure 3c). Its 20 °C reference values are 0.1, 0.5, 2, and 10 Ω, and the resistance is scaled by the cable temperature factor *k*_T_. This continuous progression represents an incipient resistive defect through a quasi-open transmission path, rather than an ideal zero-current switch.

(4) Insulation-path deterioration

A parallel RC branch is connected from phase A to ground to represent changes in the dielectric path (Figure 3d). The four paired parameter levels are *R*_ins_/*C_d_* = 100 kΩ/0.05 μF, 50 kΩ/0.10 μF, 20 kΩ/0.20 μF, and 5 kΩ/0.50 μF. Decreasing resistance and increasing capacitance produce progressively stronger power-frequency leakage and phase-path disturbance. This equivalent branch characterizes insulation deterioration through terminal-scale quantities; it does not represent individual kHz–MHz discharge pulses, pulse statistics, or apparent charge.

For each fault class, four severity levels are combined with three locations and three load torques, yielding 36 cases. Source-voltage unbalance (0%, 1%, and 2%) and cable temperature (20, 40, 60, and 80 °C) are assigned cyclically across these fault cases to obtain balanced marginal counts without expanding the design into a full six-factor combination. The 36 normal cases comprise the complete 3 × 3 × 4 combination of load, source-unbalance, and temperature levels. The resulting dataset therefore contains 180 simulation cases generated in separate runs, as summarized in Table 2.

### 3.3. Terminal Feature Extraction

Synchronized three-phase voltages and currents recorded at the source and load terminals are analyzed in the pre-fault interval of 1.2–1.4 s and the post-fault interval of 2.2–2.4 s. Each 0.2 s window contains ten cycles at 50 Hz. The fundamental RMS phasor of each channel is obtained by complex-exponential projection at 50 Hz, after which the positive-, negative-, and zero-sequence components are calculated by the symmetrical-component transformation.

Five terminal indicators are calculated from the sequence components (Table 3): the load-terminal voltage-unbalance factor, the source-terminal current-unbalance factor, the source-terminal zero-sequence current ratio, the positive-sequence voltage attenuation between the two terminals, and the corresponding phase deviation. Together, these quantities describe phase asymmetry, the presence of a ground-return path, and the magnitude and phase changes accumulated along the cable.

To suppress differences in the pre-fault operating point, each indicator *q* is expressed as a within-case change, Δ*q* = *q*_post_ − *q*_pre_. Phase differences are wrapped to the interval [−180°, 180°]. This paired-window representation preserves the fault-induced change while reducing sensitivity to the absolute baseline established by load torque, source imbalance, and cable temperature.

## 4. Terminal-Response Characteristics and Physics-Guided Diagnosis

The fault modules and paired-window indicators defined in Section 3 provide a common basis for examining how distinct impedance paths appear at the accessible terminals. This section first establishes the representative time-domain responses, then evaluates the complete 180-case feature distributions and their dependence on severity, position, and motor load. The resulting physical patterns are subsequently incorporated into a categorical fuzzy diagnostic framework and evaluated by operating-condition-grouped validation. All 180 records contained 25,001 synchronized samples over 0–2.5 s, with no nonfinite values or incomplete channels; therefore, no case was excluded from the statistical analysis.

### 4.1. Terminal Responses Under Normal and Fault Conditions

#### 4.1.1. Normal Operating Condition

**Figure 4:**
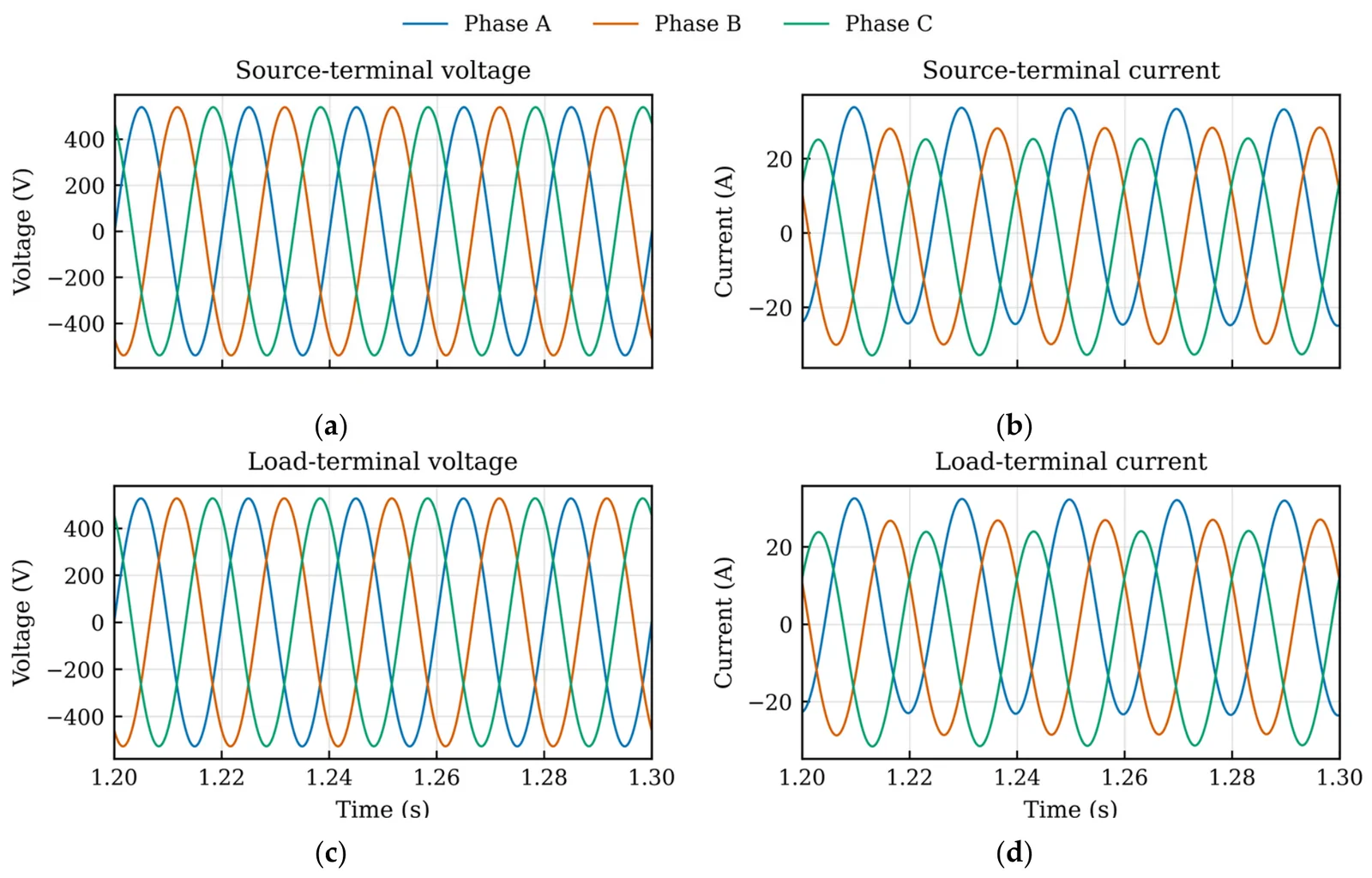
Representative three-phase voltage and current waveforms under normal operation: (**a**) source-terminal voltage; (**b**) source-terminal current; (**c**) load-terminal voltage; and (**d**) load-terminal current.

Before comparing the fault-dependent responses, the normal terminal behavior was examined under a representative operating condition. Figure 4 shows the source- and load-terminal three-phase voltage and current waveforms at a motor load torque of 250 N·m, balanced source voltage, and a cable temperature of 60 °C. The phase sequences remain regular at both terminals, while the modest source-load differences reflect the distributed cable and load impedances rather than an inserted fault path.

The 36 normal cases cover the complete combination of three motor torques, three source-unbalance levels, and four cable temperatures. Their paired-window changes remain concentrated near zero: the median load-terminal voltage-unbalance change is 4.70 × 10^−5^%, the median zero-sequence current-ratio change is −7.50 × 10^−5^%, and the median attenuation change is 0.0168%. The narrow ranges in Table 4 establish the within-run drift expected when no fault module is activated. Because each source imbalance is maintained in both analysis windows, its baseline contribution is removed by the pre-fault/post-fault difference rather than being interpreted as a new fault signature. These cases represent distinct steady operating points; within each run, motor load, source-voltage unbalance, and cable temperature remain fixed between the paired analysis windows. Consequently, the normal set characterizes baseline variation across steady conditions but does not emulate non-fault operating changes occurring between the two windows.

**Figure 5:**
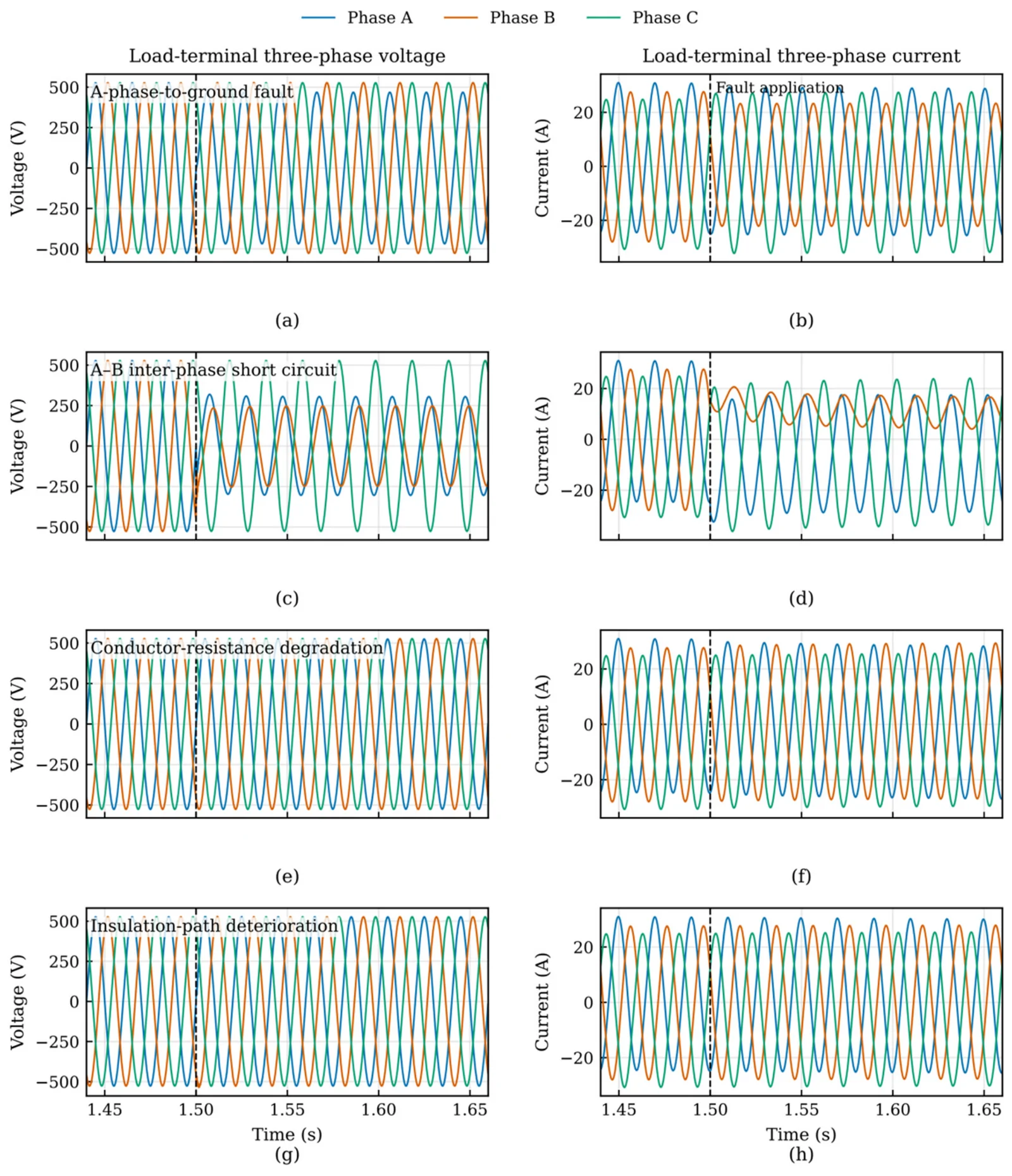
Representative load-terminal three-phase voltage and current waveforms for the four modeled cable conditions: (**a**,**b**) A-phase-to-ground fault; (**c**,**d**) A–B inter-phase short circuit; (**e**,**f**) conductor-resistance degradation; and (**g**,**h**) insulation-path deterioration.

To isolate the fault-mechanism-dependent response, Figure 5 compares the four modeled fault classes at severity level 2, a normalized fault position of *x*_f_ = 0.50, a motor load torque of 250 N·m, 0% source-voltage unbalance, and a cable temperature of 60 °C. The voltage and current responses are presented in paired columns, and the dashed vertical line denotes fault application at 1.5 s. This common-condition comparison provides the time-domain basis for the class-specific feature patterns discussed in Section 4.1.2, Section 4.1.3, Section 4.1.4 and Section 4.1.5.

#### 4.1.2. Phase-to-Ground Fault Response

As shown in Figure 5a,b, connecting phase A to ground produces the largest zero-sequence response in the case set. Across the 36 phase-to-ground cases, the source-terminal zero-sequence current-ratio change ranges from 84.51% to 95.44%, while the load-terminal voltage-unbalance change ranges from 5.88% to 37.17%. Their simultaneous increase reflects current returning through the ground path and the associated redistribution of phase voltages. Voltage attenuation also increases, although its broader 1.55–23.74% range makes it a secondary indicator for this class.

#### 4.1.3. Inter-Phase Short-Circuit Response

Figure 5c,d shows that the A–B conductive bridge produces a response pattern distinct from that of the ground-referenced fault. The median load-terminal voltage-unbalance change reaches 69.48%, and the median source–load attenuation change reaches 40.08%, whereas the zero-sequence current-ratio change remains close to zero. This combination is consistent with strong inter-phase equalization without a ground-return path and demonstrates why voltage asymmetry and attenuation must be interpreted jointly with the zero-sequence quantity.

#### 4.1.4. Local Conductor-Resistance Degradation

As shown in Figure 5e,f, the additional phase-A series resistance produces a gradual rather than abrupt change in the terminal state. Across the 36 conductor-degradation cases, the voltage-unbalance change spans 0.0359–18.92% and the attenuation change spans 0.0180–8.18%, with both increasing as the inserted resistance rises. The lowest severity level remains close to the normal envelope, whereas the higher levels become progressively separated. This response is consistent with an increasing phase-specific voltage drop and establishes the most gradual class boundary used in the diagnostic model.

#### 4.1.5. Insulation-Path Deterioration

Figure 5g,h shows that the phase-to-ground RC branch produces a weak time-domain disturbance at the illustrated severity. Across the complete class, the source-terminal zero-sequence current-ratio change increases from 0.00412% to 0.14962%, while voltage unbalance and attenuation remain close to their normal ranges. The class is therefore distinguished primarily by its low-amplitude ground-referenced leakage response.

**Figure 6:**
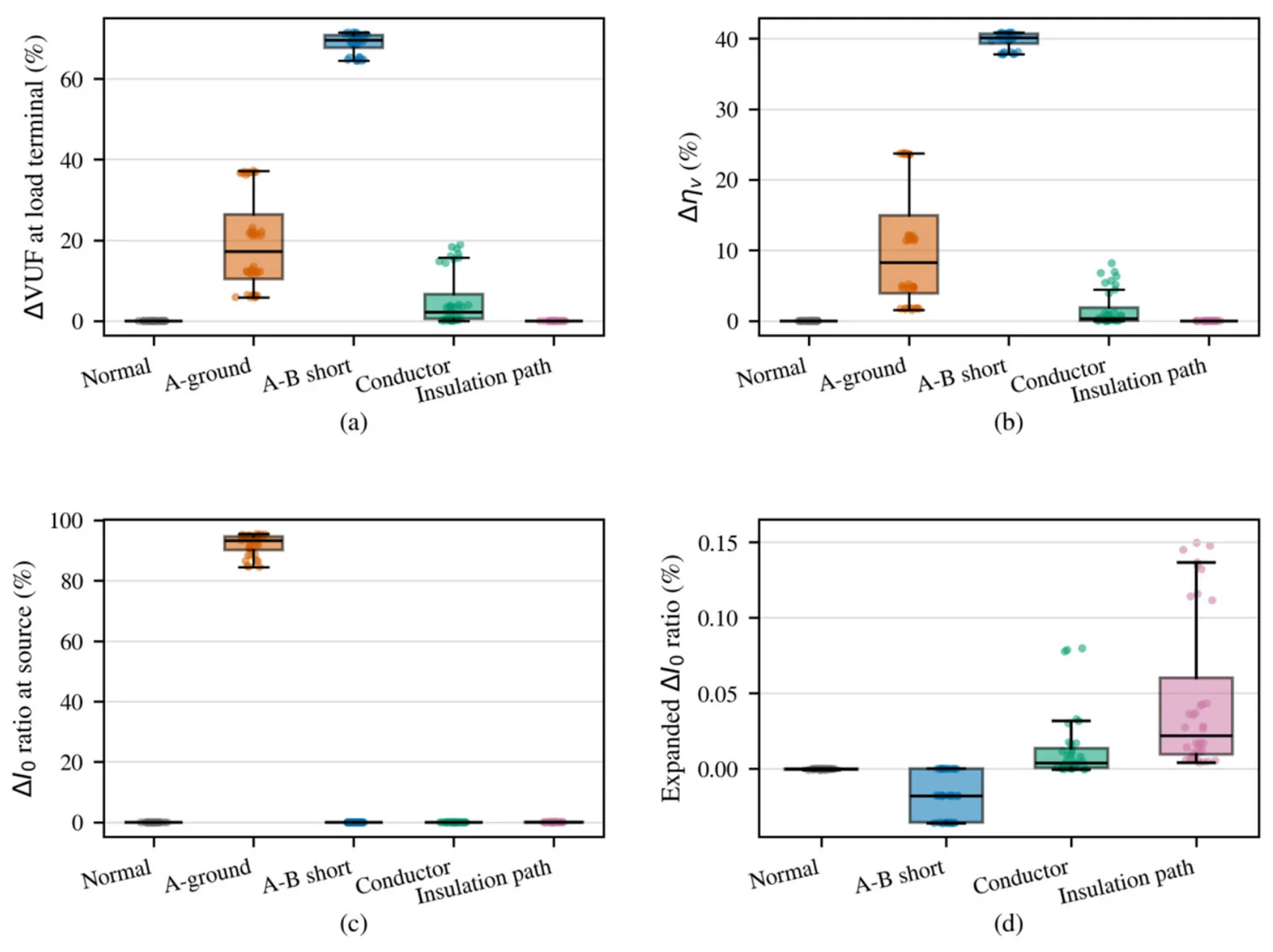
Class-wise distributions of the paired-window terminal features: (**a**) load-terminal voltage-unbalance change; (**b**) source-load attenuation change; (**c**) source-terminal zero-sequence current-ratio change over the full range; and (**d**) expanded zero-sequence range excluding the phase-to-ground class.

Taken together, the class-wise distributions of the paired-window diagnostic features are summarized in Figure 6.

The voltage-unbalance and attenuation changes provide clear separation for the A–B short-circuit class, the zero-sequence current-ratio change identifies the ground-referenced fault path, and the expanded low-amplitude range reveals the gradual overlap among normal operation, conductor degradation, and insulation-path deterioration.

### 4.2. Response Trends Across Fault Severity, Fault Position, and Motor Load

Because source imbalance and cable temperature were assigned cyclically rather than fully crossed in the fault cases, the following curves describe trends across the sampled operating design rather than fully isolated factorial effects of severity or fault position.

**Figure 7:**
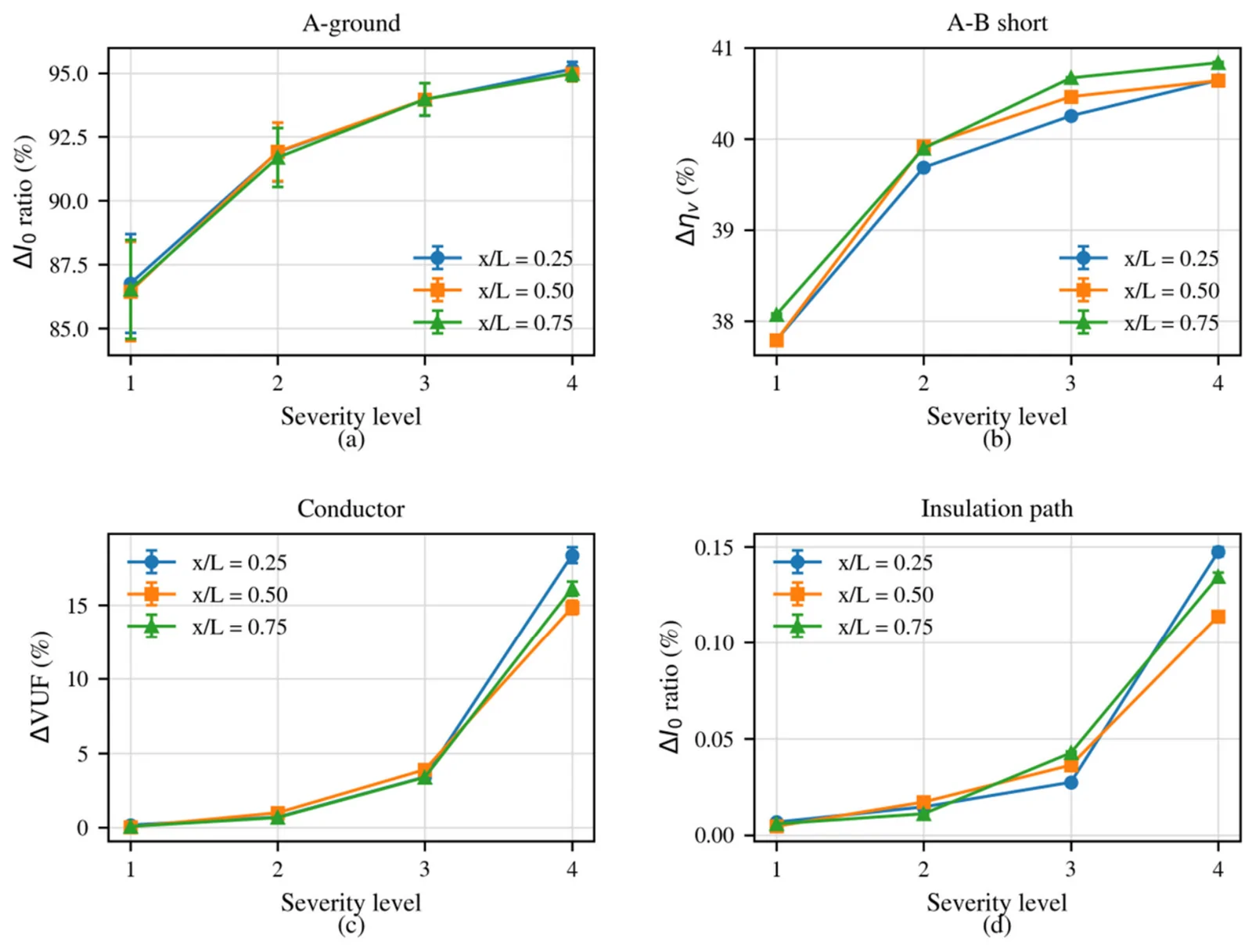
Severity-dependent dominant features at the three normalized fault locations: (**a**) A-phase-to-ground fault; (**b**) A–B inter-phase short circuit; (**c**) conductor-resistance degradation; and (**d**) insulation-path deterioration.

Figure 7 summarizes the dominant mechanism-related feature as a function of fault severity and normalized fault position, with the points and error bars denoting the mean and standard deviation across the three motor-load cases. For the phase-to-ground cases, the mean zero-sequence current-ratio change increases from 86.57% at severity level 1 to 95.03% at level 4. The attenuation change associated with the A–B inter-phase short circuit increases from 37.88% to 40.71%. The strongest severity-dependent progression occurs for conductor-resistance degradation, for which the mean voltage-unbalance change increases from 0.1008% to 16.43%. The zero-sequence current-ratio change associated with insulation-path deterioration similarly increases from 0.0057% to 0.1319%. Across the sampled fault positions, the mechanism-dependent ordering remains visible, although the response magnitude varies, particularly for high-severity conductor-resistance degradation.

**Figure 8:**
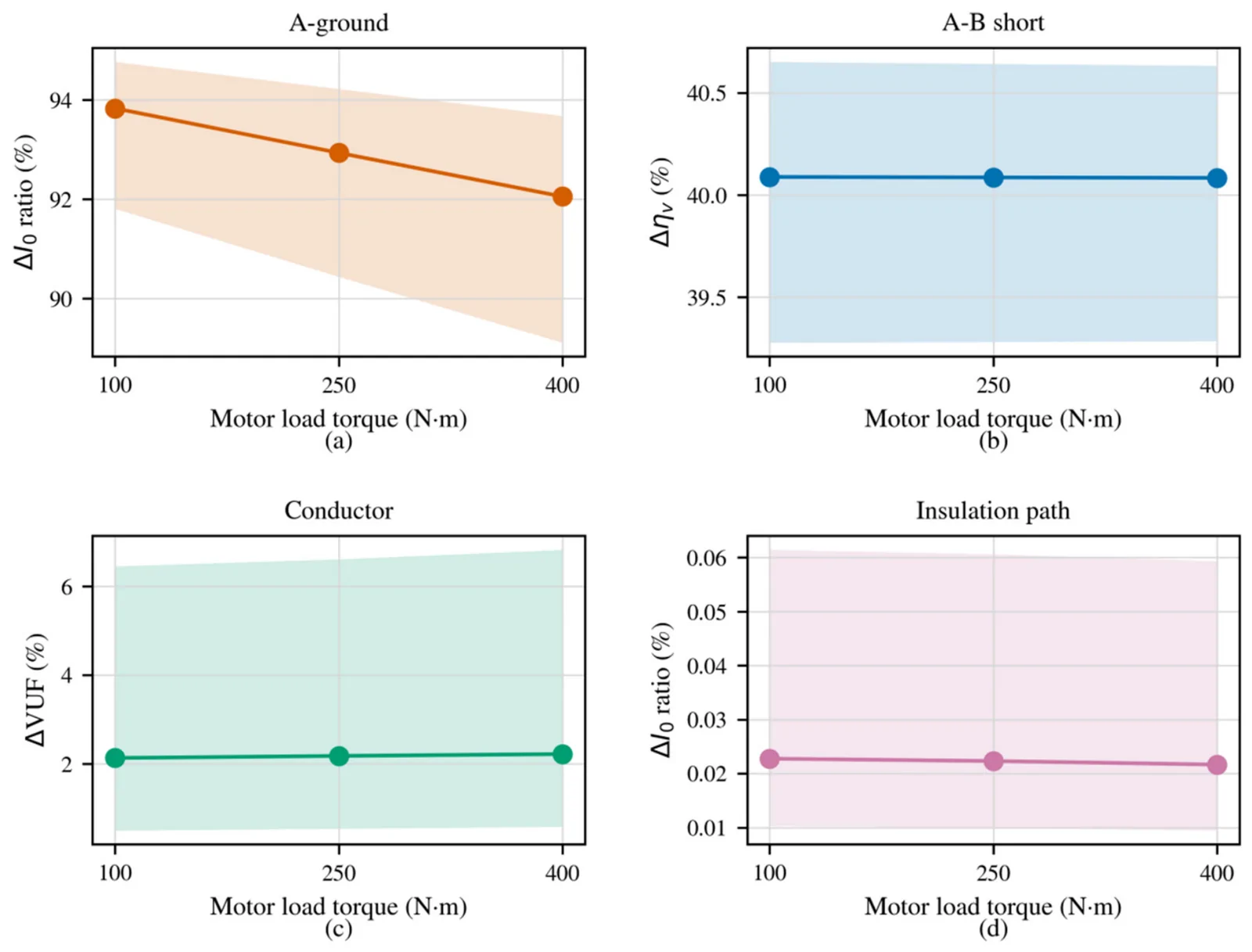
Dependence of the dominant terminal feature on motor load: (**a**) A-phase-to-ground fault; (**b**) A–B inter-phase short circuit; (**c**) conductor-resistance degradation; and (**d**) insulation-path deterioration.

Motor loading affects the absolute transmission state, but the principal class signatures remain stable across 100, 250, and 400 N·m. As shown in Figure 8, the median ground-fault zero-sequence change decreases modestly from 93.83% to 92.05%, the short-circuit attenuation remains approximately 40.09%, and the conductor voltage-unbalance change varies from 2.13% to 2.22%. The insulation-path zero-sequence change remains within 0.0217–0.0228%. Source imbalance and temperature are distributed across the fault cases as specified in Table 2, while their complete factorial combination is represented in the normal class. Their combined influence is subsequently controlled through operating-condition-grouped validation rather than treated as additional independent fault samples.

### 4.3. Measurement-Informed Support for Model Parameters

**Figure 9:**
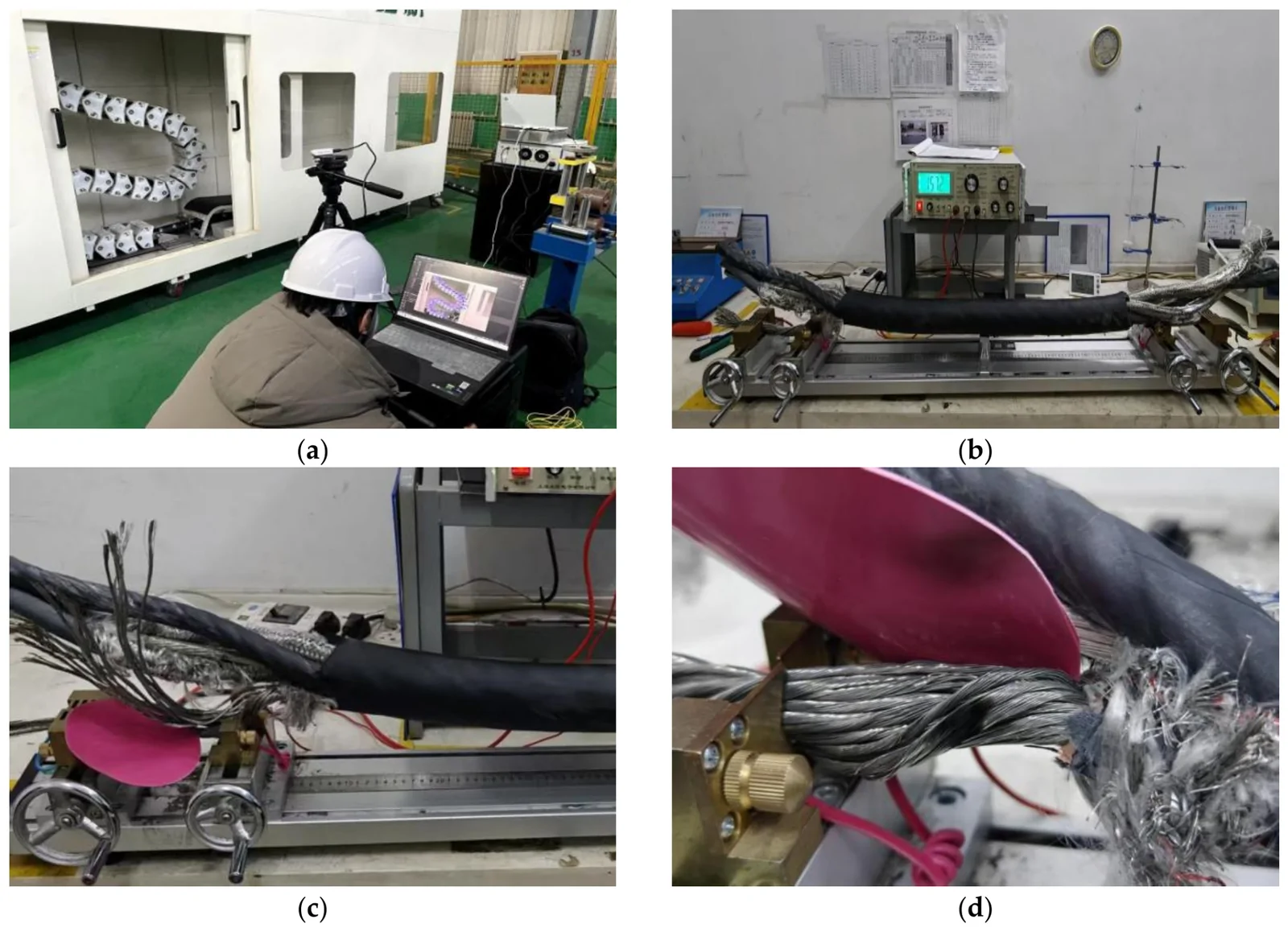
Measurement-informed parameter support: (**a**) geometric measurement; (**b**) DC-resistance setup at controlled temperature; (**c**) progressive strand interruption; and (**d**) localized thermal exposure used as an auxiliary resistance-drift check.

The laboratory measurements support the specification of cable geometry and the physically admissible progression of conductor resistance, whereas diagnostic performance is evaluated using the simulated terminal responses. The DC resistance measurements were performed using a PC36C DC resistance tester (Shanghai Taiou Electronics Co., Ltd., Shanghai, China) at the Yankuang Changlong Cable Factory in Shandong Province, China. Figure 9a documents the cable structure and dimensions, Figure 9b,c present the controlled-temperature DC-resistance measurements, and Figure 9d illustrates the localized thermal-exposure procedure. The measurements comprise geometric inspection, controlled-temperature resistance tests, and localized thermal exposure; they do not include energized inter-phase faults or high-frequency discharge measurements.

**Figure 10:**
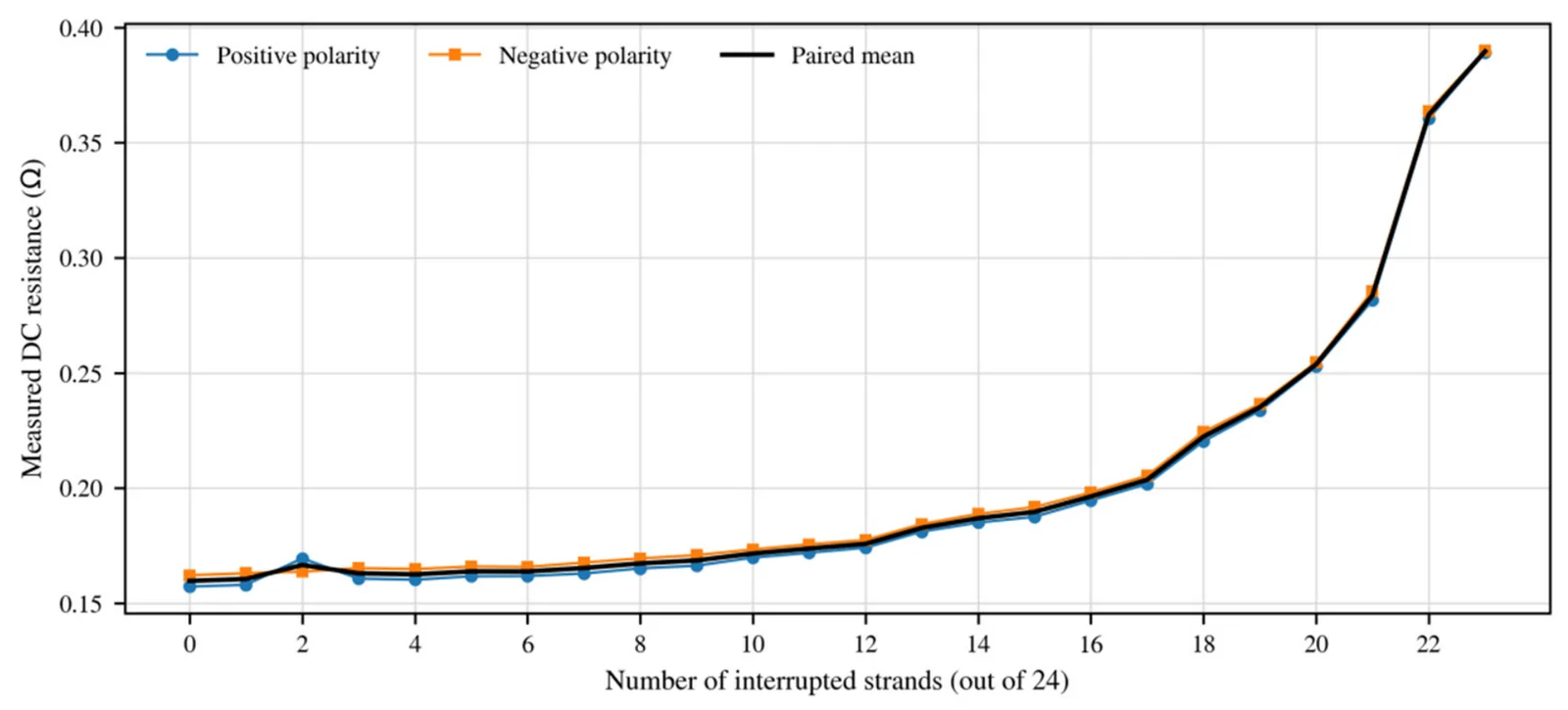
DC-resistance evolution during progressive strand interruption.

For each of the 24 strand states, resistance was recorded with positive and negative terminal polarities. These paired readings are retained as a consistency check rather than described as independent repeated trials. The polarity difference ranges from 0.0009 to 0.0057 Ω. As shown in Figure 10, the paired mean increases from 0.1598 Ω for the intact conductor to 0.3897 Ω after interruption of 23 strands, corresponding to an increase of approximately 144%. The measured trend supports the direction and order of the series-resistance progression used for the conductor-degradation module, while the diagnostic performance reported below is evaluated using held-out simulated operating groups.

### 4.4. Physics-Guided Categorical Fuzzy Diagnosis

#### 4.4.1. Feature Selection, Membership Construction, and Diagnostic Rules

The class-wise feature distributions in Figure 6 identify three complementary diagnostic quantities. The load-terminal voltage-unbalance-factor change ΔVUF_load_ responds strongly to inter-phase conduction and conductor-resistance degradation; the source-terminal zero-sequence current-ratio change Δ*I*_0,source_ distinguishes ground-referenced leakage paths; and the source-to-load voltage-attenuation change Δ*η_v_* reinforces the inter-phase short-circuit response. These quantities form the diagnostic vector **x** = [ΔVUF_load_, Δ*I*_0,source_, Δ*η_v_*]. Source-terminal current unbalance and voltage phase deviation remain useful for waveform interpretation but are not included in the classifier because their class distributions exhibit greater overlap. The selected feature vector is physically applicable to shearer power-core cable systems with a comparable three-phase source–cable–load structure and is supported by both the response mechanism of each fault path and the class separation observed across the 180 simulated cases. For different cable specifications, the feature definitions and diagnostic rule structure remain applicable, whereas the numerical membership parameters can be recalibrated using the corresponding operating data.

**Figure 11:**
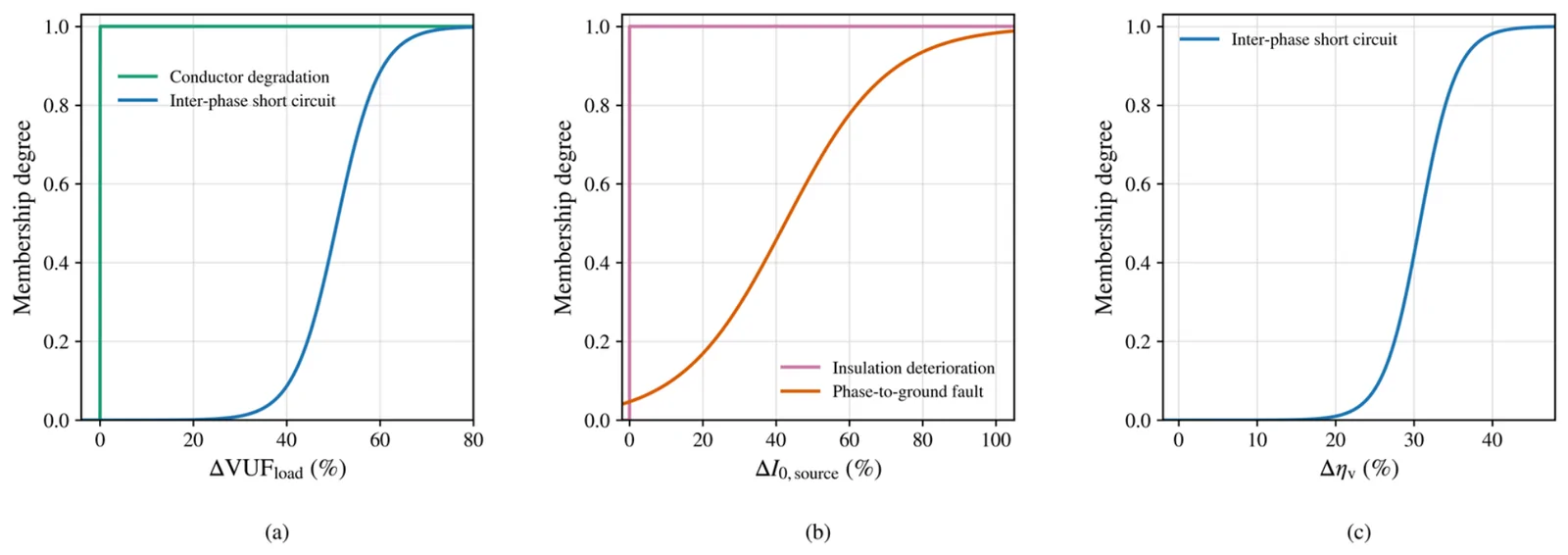
Training-derived rising membership functions associated with (**a**) load-terminal voltage-unbalance-factor change, (**b**) source-terminal zero-sequence current-ratio change, and (**c**) source-to-load voltage-attenuation change.

Within each repeated grouped-holdout evaluation, the membership parameters are estimated exclusively from the training data. Each rising state is represented by the logistic function

(14) $$\mu (x)=\frac{1}{1+\exp\left[-\frac{x-\theta }{w}\right]}$$

where *θ* is the feature value at a membership degree of 0.5 and *w* controls the transition width. For each physically relevant transition, *θ* is placed midway between the largest training value in the lower-response class and the smallest training value in the higher-response class, while *w* is set to one-sixth of this separation. Table 5 reports the mean and standard deviation of the parameters obtained from the 20 repeated evaluations, and Figure 11 shows the corresponding rising membership functions. The complementary low-response state is defined as 1 − *μ*(*x*).

The labels in Table 5 denote fault-associated linguistic response states and should not be interpreted as fault-severity grades. The conductor-degradation response denotes an increase in ΔVUF_load_ caused by the phase-specific voltage drop accompanying conductor-resistance growth. The inter-phase short-circuit response denotes the simultaneous large increases in ΔVUF_load_ and Δ*η_v_*. The insulation-leakage response represents the small but detectable increase in Δ*I*_0,source_ produced by the equivalent deteriorated insulation path, whereas the phase-to-ground response represents the substantially larger zero-sequence current associated with a dominant ground-return path.

The conductor-degradation transition occurs at the comparatively small ΔVUF_load_ threshold of 0.025 ± 0.005%, enabling low-amplitude phase-resistance growth to be represented. In contrast, the inter-phase short-circuit transitions occur at 50.765 ± 0.060% for ΔVUF_load_ and 30.759 ± 0.004% for Δ*η_v_*, reflecting the pronounced voltage asymmetry and attenuation produced by the conductive bridge. The two zero-sequence-current transitions distinguish the weak insulation-leakage response from the much larger phase-to-ground response.

The diagnostic rules combine these fault-associated states according to the physical hierarchy of the modeled current paths. A phase-to-ground fault is assigned when the ground-return membership is dominant. An A–B inter-phase short circuit requires simultaneous high memberships in the voltage-unbalance and attenuation responses associated with inter-phase conduction, while the ground-return membership remains low. Conductor-resistance degradation is identified when the voltage-unbalance response exceeds its conductor-degradation transition but remains below the inter-phase short-circuit transition, with no dominant ground-return component. Insulation-path deterioration is assigned when the weak insulation-leakage response is activated while the conductor-degradation and ground-return responses remain low. Cases for which none of the fault-associated states are activated are assigned to normal operation. This hierarchy links each class decision to a specific conductive or dielectric path and prevents any single high-magnitude feature from determining all fault labels.

Logical conjunction is implemented by the minimum operator, negation by one minus the membership degree, and multiple rule activations assigned to the same class by the maximum operator. Because the output comprises five discrete diagnostic categories rather than a continuous control quantity, centroid defuzzification is not applied. The predicted class is the category with the greatest aggregated activation. Diagnostic confidence is calculated as the dominant class activation divided by the sum of the five class activations. The resulting procedure is therefore a categorical Mamdani-style max–min inference scheme. The confidence value expresses the relative dominance of the selected rule set; it is not interpreted as a calibrated probability or as a complete measure of sensor uncertainty.

#### 4.4.2. Grouped Holdout Evaluation and Baseline Comparison

Within-model generalization across withheld simulated operating conditions is evaluated by grouping the 180 cases according to the common combination of motor load torque, source-voltage unbalance, and cable temperature. Twenty repeated holdout evaluations allocate 70% of these operating-condition groups to training and 30% to testing. All cases sharing the same base operating condition are retained in the same partition, thereby preventing condition-level information leakage. All random operations used MATLAB’s Mersenne Twister generator. For grouped-holdout repetition r, the seed was defined as $s_{\mathrm{split}}\;=\;20260831\;+\;r$, where r = 1, …, 20. For realization j of case c at noise-level index k, the waveform-noise seed was defined as $s_{\mathrm{noise}}\;=\;20260831\;+\;100000c\;+\;1000k\;+\;j$. Thus, both the grouped partitions and waveform-noise realizations are exactly reproducible. The resulting test partitions contain 47–67 cases (mean 56.4), and all thresholds, membership parameters, standardization coefficients, and classifier parameters are estimated from the noise-free training data only. Four methods are evaluated using the same three input features: a shallow decision tree limited to four binary splits, a radial-basis-function support vector machine (RBF-SVM) implemented through one-versus-one multiclass coding, a five-nearest-neighbor classifier (k-NN) using standardized Euclidean distance, and the proposed categorical fuzzy method. Since feature selection and rule development draw on the same simulation framework, this comparison assesses the performance of the resulting diagnostic schemes on withheld simulated operating-condition combinations.

Measurement error is emulated directly in the simulated terminal voltage and current waveforms rather than by perturbing the extracted feature values. Additive zero-mean Gaussian noise comprises an independent channel component and a three-phase common component with a correlation coefficient of 0.3. A channel-wise constant multiplicative gain error is also included, with a standard deviation equal to 0.2 times the prescribed noise fraction. For every held-out case, 20 independent realizations are generated at root-mean-square (RMS) noise levels of 0.5%, 1%, and 2% relative to the channel RMS value over the analysis windows. The three diagnostic features are then recalculated from the noisy pre-fault and post-fault waveforms. For each holdout, predictions from the 20 noise realizations are pooled to calculate accuracy and macro-F1. Means and standard deviations are calculated across the 20 grouped holdout repetitions. To describe the variability caused by repeated data partitioning without assuming statistically independent experiments, the reported 95% repeated-split variability intervals are defined by the 2.5th and 97.5th percentiles of the 20 values.

Table 6 summarizes the repeated grouped-holdout results. For noise-free held-out cases, both the proposed fuzzy method and the deterministic threshold tree achieve a mean accuracy and macro-F1 of 99.80%. The RBF-SVM yields 97.36% accuracy and 97.30% macro-F1, whereas k-NN yields 94.00% and 93.90%, respectively. The identical noise-free performance of the fuzzy method and threshold tree shows that the proposed fuzzy representation preserves hard-label classification accuracy. Its additional output is the continuous activation pattern, which indicates boundary proximity while retaining a direct physical interpretation of each rule.

The waveform-level noise analysis produces a different ranking. At 1% RMS noise, the fuzzy method and threshold tree retain 81.12% mean accuracy, k-NN reaches 81.31%, and the RBF-SVM decreases to 55.83%. At 2% RMS noise, k-NN provides the highest mean accuracy of 77.07%, followed by 74.71% for the fuzzy and threshold methods and 48.68% for the RBF-SVM.

**Figure 12:**
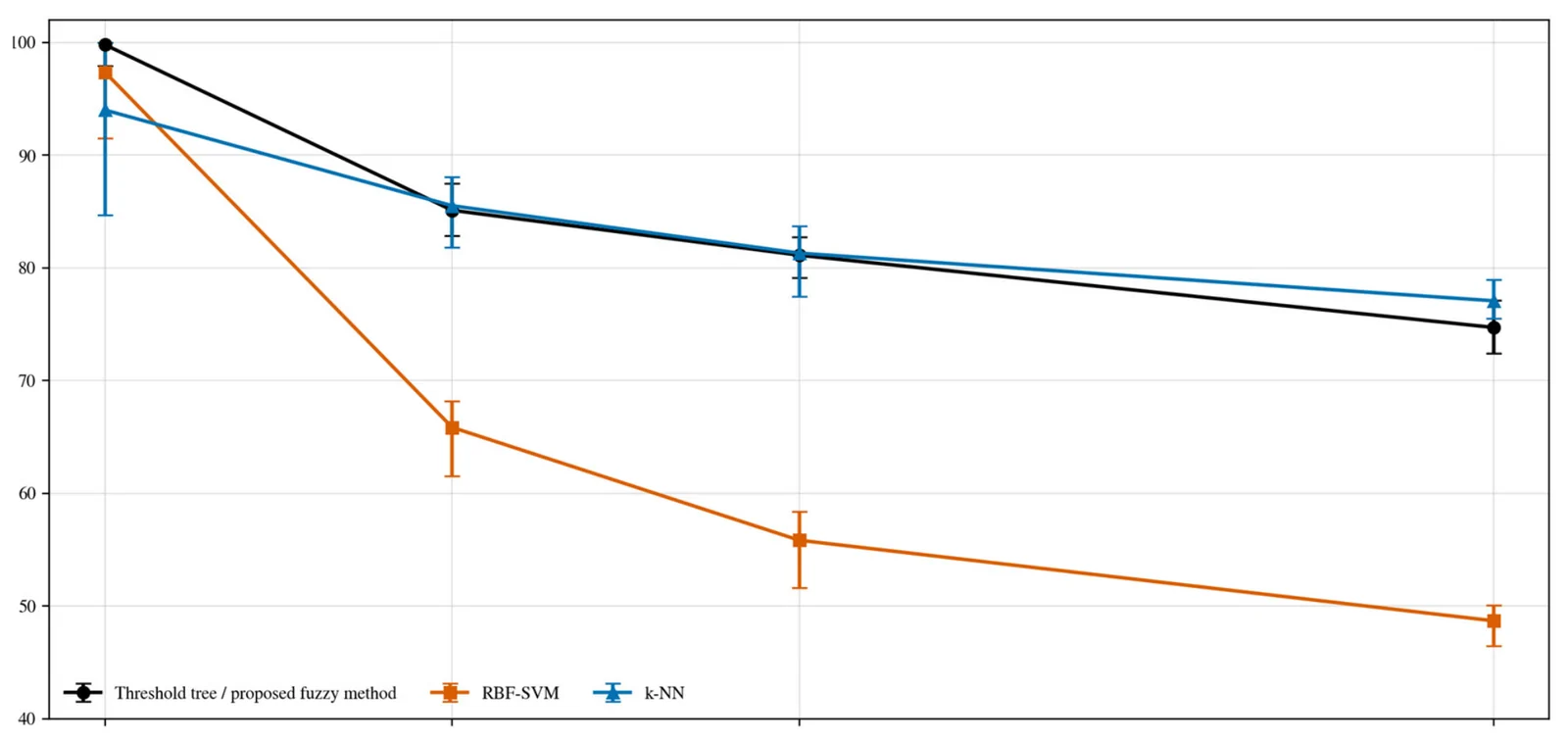
Mean classification accuracy and 95% repeated-split variability intervals over 20 operating-condition-grouped holdout evaluations.

Figure 12 shows the corresponding trends and 95% repeated-split variability intervals. At the higher noise levels, k-NN retains a modest accuracy advantage, whereas the proposed method preserves competitive hard-label performance and directly traceable class activations. The comparison demonstrates that the fuzzy framework retains transparent, physically interpretable decision logic without sacrificing noise-free accuracy relative to the matched deterministic threshold tree.

**Figure 13:**
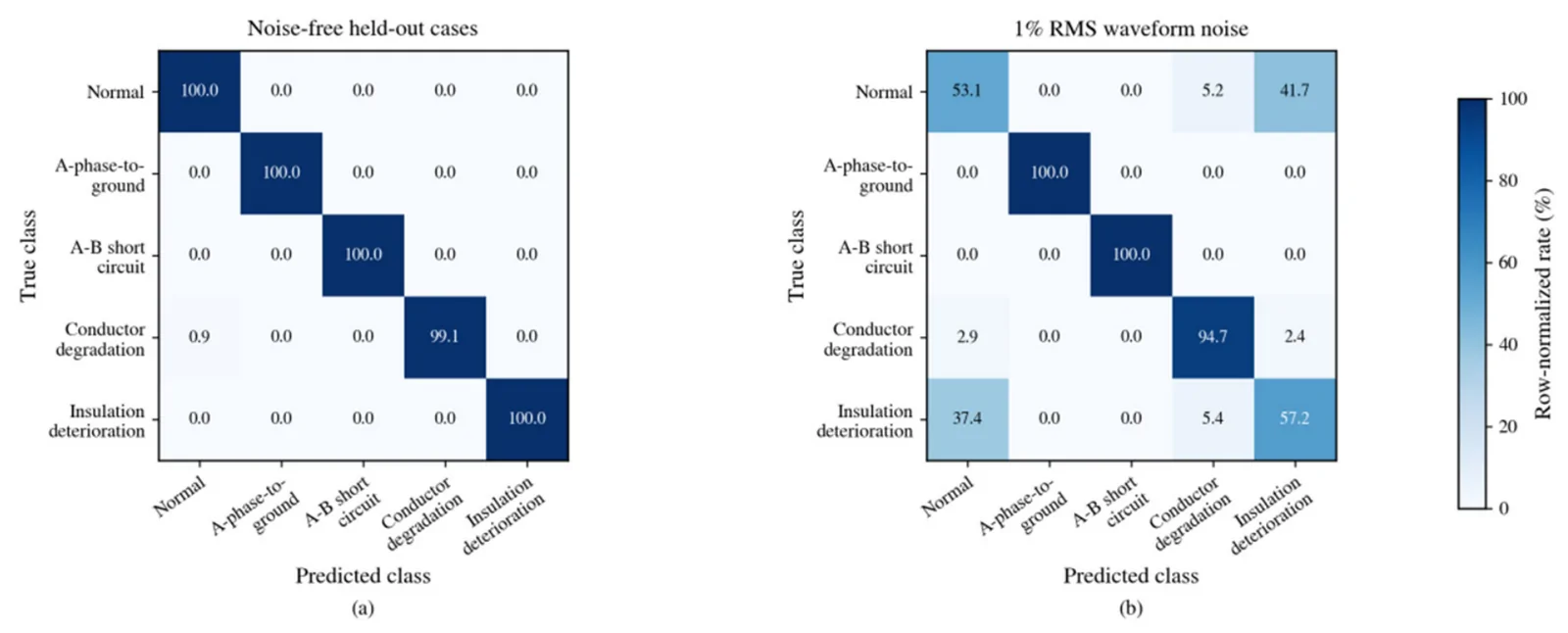
Aggregated row-normalized confusion matrices for the proposed fuzzy method: (**a**) noise-free held-out cases and (**b**) held-out cases with 1% RMS waveform noise.

Figure 13 presents the class-specific classification performance and misclassification patterns of the proposed fuzzy method. Under noise-free conditions, normal operation, phase-to-ground faults, A–B inter-phase short circuits, and insulation-path deterioration achieve 100% recall, whereas 0.9% of the conductor-resistance-degradation cases are classified as normal operation. At 1% RMS waveform noise, the phase-to-ground and inter-phase short-circuit classes remain correctly distinguished, and the recall of conductor-resistance degradation is 94.7%. The remaining errors are concentrated between normal operation and insulation-path deterioration: 41.7% of the normal cases are classified as insulation-path deterioration, while 37.4% of the insulation-path-deterioration cases are classified as normal operation. The insulation-leakage membership midpoint is 0.002176% (Table 5), while the maximum absolute normal-class variation in the source-terminal zero-sequence current-ratio change is 0.00075% (Table 4). Both quantities lie in a low-amplitude region where waveform noise can substantially affect membership activation, providing a physical explanation for the reciprocal confusion between normal operation and insulation-path deterioration in Figure 13b, consistent with the feature distributions in Figure 6d. By contrast, the stronger ground-return and inter-phase fault responses remain clearly separated.

#### 4.4.3. Severity-Dependent Performance and Diagnostic Interpretation

To determine whether the noise-induced errors are concentrated at incipient fault levels, Table 7 reports the recall of the proposed fuzzy method for each class and severity under 1% RMS waveform noise.

As summarized in Table 7, all severity levels of the phase-to-ground and A–B inter-phase short-circuit classes retain 100% recall at 1% RMS noise because their dominant features remain well separated from the normal range. Recall for conductor-resistance degradation is 77.93% at severity level 1 and reaches 100% at levels 2–4. Insulation-path-deterioration recall is 42.48% and 47.67% at severity levels 1 and 2, respectively, and increases to 60.40% and 77.72% at levels 3 and 4 as the zero-sequence response becomes stronger. Normal-operation recall is 53.07%; most false alarms are assigned to insulation-path deterioration, with a smaller proportion assigned to conductor-resistance degradation. The dominant false-alarm boundary is therefore formed by normal operation, low-severity insulation-path deterioration, and incipient conductor degradation. This result motivates repeated-window confirmation and waveform denoising as practical extensions for reducing false alarms.

The continuous rule activations provide decision information that is not available from the threshold tree’s hard label. For illustration, a low-severity conductor-resistance-degradation case withheld from training, with the resistance increase located at the normalized cable position 0.50, a motor load torque of 100 N·m, 2% source-voltage unbalance, and a cable temperature of 40 °C, produces the two largest activation degrees of 0.714 for conductor-resistance degradation and 0.286 for normal operation. Smaller nonzero activations of the remaining rule sets increase the total activation to approximately 1.119. The dominant activation therefore corresponds to a normalized confidence of 0.638, indicating proximity to the normal/conductor-degradation boundary, whereas the threshold tree returns only the categorical label. Across the noise-free grouped partitions, the mean confidence is 0.936 ± 0.008.

The additional diagnostic value of the continuous activations is quantified by applying a fixed descriptive confidence criterion of c ≥ 0.8, which is not optimized for classification accuracy. Across the 20 repeated grouped partitions, this criterion retains 99.16%, 95.92%, 92.91%, and 92.24% of predictions at RMS noise levels of 0%, 0.5%, 1%, and 2%, respectively. The corresponding conditional accuracies are 100.00%, 86.39%, 83.98%, and 78.48%, compared with overall accuracies of 99.80%, 85.11%, 81.12%, and 74.71%. The resulting conditional gains are 0.20, 1.28, 2.85, and 3.77 percentage points, respectively. At 1% RMS noise, the mean confidence is 0.935 for correct predictions and 0.871 for incorrect predictions. Thus, while the fuzzy representation retains the hard-label accuracy of the matched threshold tree, it additionally provides a quantitative indication of boundary proximity that can support repeated-window confirmation of less dominant decisions.

Taken together, the results show that the mechanism-dependent terminal-response patterns remain identifiable across the simulated variations in fault severity, fault position, motor load, source-voltage unbalance, and cable temperature. The grouped holdout evaluation further shows that the dominant ground-return and inter-phase responses remain identifiable in withheld simulated base-condition combinations, whereas normal operation and low-severity degradation are difficult to separate under waveform noise because they occupy a narrow low-amplitude region. The proposed framework therefore provides an application-specific diagnostic interpretation in which each class decision is linked to the conductive or dielectric path represented in the cable model.

## 5. Conclusions

This study developed an interpretable fault-diagnosis framework for shearer power-core cables based on voltage and current responses available at accessible source and load terminals. Its principal contribution is a fault path-oriented diagnostic hierarchy that links paired-window changes in load-terminal voltage unbalance, source-terminal zero-sequence current ratio, and source-to-load voltage attenuation to distinct conductive and dielectric deterioration mechanisms. A distributed-parameter source–cable–load model was used to construct 180 operating and fault cases, while laboratory measurements of cable geometry and conductor DC resistance provided physical support for model parameterization.

The simulated responses exhibited clear mechanism-dependent characteristics. Phase-to-ground faults were dominated by a large zero-sequence current response, A–B inter-phase short circuits produced simultaneous increases in voltage unbalance and attenuation, conductor-resistance degradation caused a progressive phase-specific voltage-unbalance response, and insulation-path deterioration generated a weaker ground-referenced leakage signature. These characteristics preserved their physical ordering across the investigated fault severities, positions, motor loads, source voltage imbalances, and cable temperatures, supporting the use of compact terminal features for distinguishing the modeled cable conditions.

Within-model generalization across simulated operating conditions was evaluated through 20 repeated 70/30 holdouts grouped by base operating condition, with all membership parameters and classifier settings determined exclusively from the training groups. The proposed categorical Mamdani-style method achieved a mean accuracy of 99.80% on noise-free test cases, compared with 99.80% for the deterministic threshold tree, 97.36% for the RBF-SVM, and 94.00% for k-NN. Under 1% and 2% RMS waveform noise, its mean accuracy remained 81.12% and 74.71%, respectively. At 1% noise, normal-operation recall was 53.07%, while insulation-path-deterioration recall was 42.48% and 47.67% at severity levels 1 and 2, respectively. These errors were concentrated at the narrow boundary between normal operation and weak insulation leakage; phase-to-ground and inter-phase short-circuit faults remained fully distinguished. Relative to the matched threshold tree, the fuzzy method preserved hard-label accuracy and added continuous rule activations: retaining 92.91% of predictions with confidence c ≥ 0.8 increased conditional accuracy from 81.12% to 83.98% at 1% noise.

The value of the proposed framework lies in combining systematic evaluation across withheld simulated operating-condition combinations with physically interpretable diagnostic decisions. By linking terminal-response changes to specific fault paths, the method provides a practical basis for screening shearer cable conditions when labeled fault records are scarce and decision traceability is required. Future work will extend the evaluation to additional cable lengths and distributed parameters, non-fault changes occurring between analysis windows, time-varying motor loads, switching conditions, and measured field records. Repeated-window confirmation and waveform denoising will also be investigated to further improve diagnostic precision and false-alarm control.

## Figures and Tables

**Table 1:** Main electrical and mechanical parameters of the source–cable–load model.

Component	Parameter	Value
Programmable source	Nominal line-to-line RMS voltage; frequency	660 V; 50 Hz
Transformer	Rated power; nominal voltages; frequency; connection	1 MVA; 660/660 V; 50 Hz; Yg–Yg
	Winding resistance and leakage inductance	R_1_ = R_2_ = 0.002 p.u.; Lℓ_1_ = Lℓ_2_ = 0.08 p.u.
	Magnetizing branch; zero-sequence flux-path inductance	R_m_ = 1000 p.u.; L_m_ = 1000 p.u.; L_0_ = 0.5 p.u.
Interface branch	Series resistance and inductance per phase	0.05 Ω; 1 mH
Cable	Total length	0.100 km
	Positive-/zero-sequence resistance at 20 °C; temperature coefficient	0.02/0.05 Ω/km; 0.00393 °C^−1^
	Positive-/zero-sequence inductance	1.0/0.5 mH/km
	Positive-/zero-sequence capacitance; shunt conductance	1/10 μF/km; 0 S/km
Asynchronous motor	Rotor type; nominal rating	Wound rotor; 730 kW; 660 V; 50 Hz; two pole pairs
	Electrical parameters	R_s_ = 0.035 Ω; Lℓ_s_ = 1 mH; R_r_ = 0.015 Ω; Lℓ_r_ = 1.2 mH; L_m_ = 0.06 H
	Mechanical parameters	J = 10 kg·m^2^; B = 0.005879 N·m·s
Auxiliary terminal load	Grounded-wye constant-PQ load	660 V; 50 Hz; 10 kW per phase; 0.1 kvar inductive and capacitive per phase

**Table 2:** Simulation case design and fault-severity levels.

Case Class	Severity Parameter	Case Construction	Cases
Normal operation	No inserted fault	3 loads × 3 source-unbalance levels × 4 temperatures	36
A-phase-to-ground fault	*R*_f_ = 2, 1, 0.5, 0.2 Ω	4 severities × 3 locations × 3 loads	36
A–B inter-phase short circuit	*Z*_s_ = 0.2, 0.1, 0.05, 0.02 Ω	4 severities × 3 locations × 3 loads	36
Conductor-resistance degradation	*R*_d,20_ = 0.1, 0.5, 2, 10 Ω	4 severities × 3 locations × 3 loads	36
Insulation-path deterioration	*R*_ins_/*C*_d_ = 100/0.05, 50/0.10, 20/0.20, 5/0.50 (kΩ/μF)	4 severities × 3 locations × 3 loads	36
Total	—	—	180

**Table 3:** Terminal indicators derived from the fundamental sequence components.

Indicator	Definition	Physical Interpretation
VUF at load terminal	100|*V*_2,l_|/|*V*_1,l_| (%)	Load-terminal voltage unbalance
IUF at source terminal	100|*I*_2,s_|/|*I*_1,s_| (%)	Source-terminal current unbalance
Zero-sequence current ratio	100|*I*_0,s_|/|*I*_1,s_| (%)	Phase-to-ground current path
Voltage attenuation *η*_v_	100(1 − |*V*_1,l_|/|*V*_1,s_|) (%)	Positive-sequence voltage loss along the cable
Phase deviation *φ*_v_	∠(*V*_1,l_/*V*_1,s_) (°)	Positive-sequence phase displacement between terminals

**Table 4:** Median and range [minimum, maximum] of the three diagnostic feature changes across the 180 cases.

Class	ΔVUF_load_ (%)	Δ*I*_0,source_ (%)	Δ*η*_v_ (%)
Normal operation	0.00005 [−0.00012, 0.00026]	−0.00007 [−0.00075, 0.00000]	0.017 [0.006, 0.028]
A-phase-to-ground fault	17.254 [5.877, 37.172]	93.197 [84.508, 95.441]	8.276 [1.551, 23.744]
A–B inter-phase short circuit	69.476 [64.421, 71.453]	−0.018 [−0.036, 0.000]	40.083 [37.777, 40.847]
Conductor-resistance degradation	2.146 [0.036, 18.916]	0.004 [0.000, 0.080]	0.334 [0.018, 8.183]
Insulation-path deterioration	0.00003 [−0.00216, 0.00886]	0.022 [0.004, 0.150]	0.017 [0.004, 0.036]

**Table 5:** Training-derived parameters of the rising membership functions over 20 repeated grouped-holdout evaluations (mean ± standard deviation).

Input Feature	Fault-Associated Rising State	Threshold (%)	Transition Width (%)
$\Delta {\mathrm{VUF}}_{\mathrm{load}}$	Conductor-degradation response	0.025 ± 0.005	0.006 ± 0.002
$\Delta {\mathrm{VUF}}_{\mathrm{load}}$	Inter-phase short-circuit response	50.765 ± 0.060	4.559 ± 0.023
$\Delta \eta_{v}$	Inter-phase short-circuit response	30.759 ± 0.004	2.340 ± 0.002
$\Delta I_{0,\mathrm{source}}$	Insulation-leakage response	0.002176 ± 0.000191	0.000725 ± 0.000064
$\Delta I_{0,\mathrm{source}}$	Phase-to-ground response	42.435 ± 0.291	14.095 ± 0.097

**Table 6:** Performance of the four diagnostic methods under different RMS waveform-noise levels (%).

Waveform Noise (% RMS)	Method	Accuracy, Mean ± SD (%)	95% Repeated-Split Variability Interval (%)	Macro-F1 (%)
0 (noise-free)	Deterministic threshold tree	99.80 ± 0.63	[97.95, 100.00]	99.80
	RBF-SVM	97.36 ± 3.01	[91.53, 100.00]	97.30
	k-NN	94.00 ± 4.61	[84.66, 100.00]	93.90
	Proposed fuzzy method	99.80 ± 0.63	[97.95, 100.00]	99.80
0.5	Deterministic threshold tree	85.11 ± 1.34	[82.86, 87.47]	84.98
	RBF-SVM	65.85 ± 2.26	[61.55, 68.18]	61.25
	k-NN	85.52 ± 1.99	[81.82, 88.09]	85.57
	Proposed fuzzy method	85.11 ± 1.34	[82.86, 87.47]	84.98
1.0	Deterministic threshold tree	81.12 ± 1.15	[79.13, 82.72]	80.81
	RBF-SVM	55.83 ± 1.83	[51.63, 58.38]	51.69
	k-NN	81.31 ± 1.92	[77.47, 83.69]	81.50
	Proposed fuzzy method	81.12 ± 1.15	[79.13, 82.72]	80.81
2.0	Deterministic threshold tree	74.71 ± 1.38	[72.42, 77.10]	73.44
	RBF-SVM	48.68 ± 1.14	[46.48, 50.08]	43.54
	k-NN	77.07 ± 1.13	[75.50, 78.96]	76.84
	Proposed fuzzy method	74.71 ± 1.38	[72.42, 77.10]	73.44

**Table 7:** Recall of the proposed fuzzy method by fault class and severity under 1% RMS waveform noise (%).

Class	Severity	Mean Recall (%)	SD (%)	95% Repeated-Split Variability Interval (%)
Normal operation	—	53.07	3.22	[47.66, 58.68]
A-phase-to-ground fault	1	100.00	0.00	[100.00, 100.00]
A-phase-to-ground fault	2	100.00	0.00	[100.00, 100.00]
A-phase-to-ground fault	3	100.00	0.00	[100.00, 100.00]
A-phase-to-ground fault	4	100.00	0.00	[100.00, 100.00]
A–B inter-phase short circuit	1	100.00	0.00	[100.00, 100.00]
A–B inter-phase short circuit	2	100.00	0.00	[100.00, 100.00]
A–B inter-phase short circuit	3	100.00	0.00	[100.00, 100.00]
A–B inter-phase short circuit	4	100.00	0.00	[100.00, 100.00]
Conductor-resistance degradation	1	77.93	7.42	[65.00, 87.50]
Conductor-resistance degradation	2	100.00	0.00	[100.00, 100.00]
Conductor-resistance degradation	3	100.00	0.00	[100.00, 100.00]
Conductor-resistance degradation	4	100.00	0.00	[100.00, 100.00]
Insulation-path deterioration	1	42.48	5.23	[35.00, 50.00]
Insulation-path deterioration	2	47.67	7.73	[35.59, 62.50]
Insulation-path deterioration	3	60.40	5.31	[55.00, 71.04]
Insulation-path deterioration	4	77.72	5.52	[67.90, 86.31]

## Data Availability

The data presented in this study are available on request from the corresponding author.
